# Attachment and mentalization as predictors of outcome in family therapy for adolescent anorexia nervosa

**DOI:** 10.1007/s00787-021-01930-3

**Published:** 2021-12-30

**Authors:** Tom Jewell, Moritz Herle, Lucy Serpell, Alison Eivors, Mima Simic, Peter Fonagy, Ivan Eisler

**Affiliations:** 1 Florence Nightingale Faculty of Nursing, Midwifery and Palliative Care, King’s College London, London, UK; 2 Great Ormond Street Hospital NHS Foundation Trust, London, UK; 3 Department of Biostatistics and Health Informatics, King’s College London, London, UK; 4 North East London NHS Foundation Trust, London, UK; 5 Research Department of Clinical, Educational and Health Psychology, University College London, London, UK; 6 Leicestershire Partnership NHS Foundation Trust, Leicester, UK; 7 South London and Maudsley NHS Foundation Trust, London, UK; 8 Anna Freud Centre, London, UK

**Keywords:** Attachment, Mentalization, Family therapy, Anorexia nervosa, Therapeutic alliance

## Abstract

Anorexia nervosa-focussed family therapy (FT-AN) is the first-line treatment for adolescent anorexia nervosa (AN), but the predictors of poor treatment response are not well understood. The main aim of this study was to investigate the role of attachment and mentalization in predicting treatment outcome. The secondary aims of the study were to investigate therapeutic alliance at 1 month as a predictor of outcome, and to test the associations between alliance and baseline attachment and mentalization. 192 adolescents with AN and their parents were recruited as they began family therapy in out-patient specialist eating disorder services. Self-report measures of attachment, mentalization, and emotion regulation were completed at the start of treatment by adolescent patients and one of their parents. Self-reported alliance scores were collected at one month. Higher scores on the Certainty Scale of the Reflective Functioning Questionnaire, completed by parents, which indicate over-certainty about mental states, were the strongest predictor of poor outcome (Odds Ratio: 0.42, CI: 0.20–0.87). Similarly, for adolescents, higher Lack of Clarity scores on the Difficulties in Emotion Regulation Scale, representing being unclear about one’s feelings, were predictive of positive treatment outcome (OR: 1.10, CI: 1.00–1.21). Higher alliance scores at 1 month predicted positive outcome, and were associated with attachment security and mentalization. These novel findings suggest that, particularly in parents, a tendency towards excessive certainty about mental states in others may predict poor outcome in FT-AN. Further research is warranted to replicate the finding and characterise families at risk of poor outcome.

**Supplementary Information:**

The online version contains supplementary material available at 10.1007/s00787-021-01930-3.

## Introduction

Anorexia nervosa (AN) is a serious condition which typically emerges during adolescence. AN is associated with high rates of psychiatric co-morbidity [1] and significantly raised mortality rates [2], with an estimated incidence of 14 per 100,000 [3]. Specialist anorexia nervosa-focussed family therapy for children and young people (FT-AN) has the strongest efficacy evidence in the treatment of adolescent AN and was recommended by NICE [4] as the first-line treatment. However, a significant proportion of patients fail to respond and a lack of evidence on predictors of treatment response and understanding of mechanisms of change limits efforts to personalise treatment recommendations. There is therefore an urgent need for well-designed and sufficiently powered research that can investigate predictors of response and change processes in FT-AN [5].

Currently, the most robust findings relating to treatment response in FT-AN relate to clinical indicators, with shorter duration of illness and higher percentage body mass index at start of treatment being associated with more positive outcomes (see Jewell et al. [6] for a review). In terms of early markers, early weight gain [7] and positive ratings of the therapeutic alliance are predictive of good treatment response in FT-AN [8]. In terms of relational variables, there is evidence that parental expressed emotion is associated with poorer outcome [9], but there is almost no research on how adolescent psychological characteristics beyond features of eating pathology might impact treatment outcome.

The present study sought to shed light on predictors of outcome and mechanisms of change in FT-AN by focussing on two constructs that are salient in eating disorders: attachment and mentalizing. *Attachment* can be understood as a broad, higher order construct which has been operationalized in multiple ways, with a foundation in the conceptual and empirical work of Bowlby [10] and Ainsworth [11]; *attachment style*, the operationalization used in this study, refers to a constellation of knowledge, expectations, and insecurities that people hold about themselves and their close relationships [12]. Meta-analytic evidence suggests that individuals with eating disorders have higher rates of insecure attachment, and more difficulties mentalizing, than community controls [6, 13]. Moreover, attachment is an established predictor of both outcome and therapeutic alliance in adult psychotherapy [14, 15]. In adult eating disorders, attachment security has been shown to predict positive therapeutic alliance [16] and differential response to treatment [17], including rates of dropout.

*Mentalization* refers to the capacity to understand others’ actions as well as one’s own behaviour in terms of intentional mental states, such as feelings, desires, attitudes, and goals, and is assumed to predict the capacity for emotion regulation. *Reflective function* refers to this ability operationalized in the context of attachment relationships [18]. Difficulties in reflective function can be conceptualised as falling into two broad domains: *hypomentalizing* refers to an inability to consider complex models of one’s own mind and/or that of others, whereas *hypermentalizing* involves making unjustified assumptions about other people’s mental states that go far beyond the observable data [19]. Mentalization has been shown to predict alliance [20] and outcomes [21] in adults with eating disorders.

### Proposed links between attachment, mentalization, and treatment outcome

A person’s ability to mentalize effectively is a dynamic capacity that is influenced by stress and arousal, particularly in the context of attachment relationships [19]. Under conditions of high arousal, patterns of brain activity ‘switch’ from flexibility to automaticity [22], leading to *pre-mentalizing modes* of experiencing subjectivity, such as over-certain modes of thought [23]. Attachment security, which raises the threshold for this switch-point to automatic/pre-mentalizing modes of thought, can thus be conceptualised as a protective factor for individuals experiencing affectively charged occurrences within their attachment network. We therefore hypothesised that attachment security and capacity for mentalization would be associated with positive treatment outcome in FT-AN, given that the process of attending treatment for, and recovering from, AN is associated with high levels of stress for adolescents and their families [24].

**Fig. 1:**
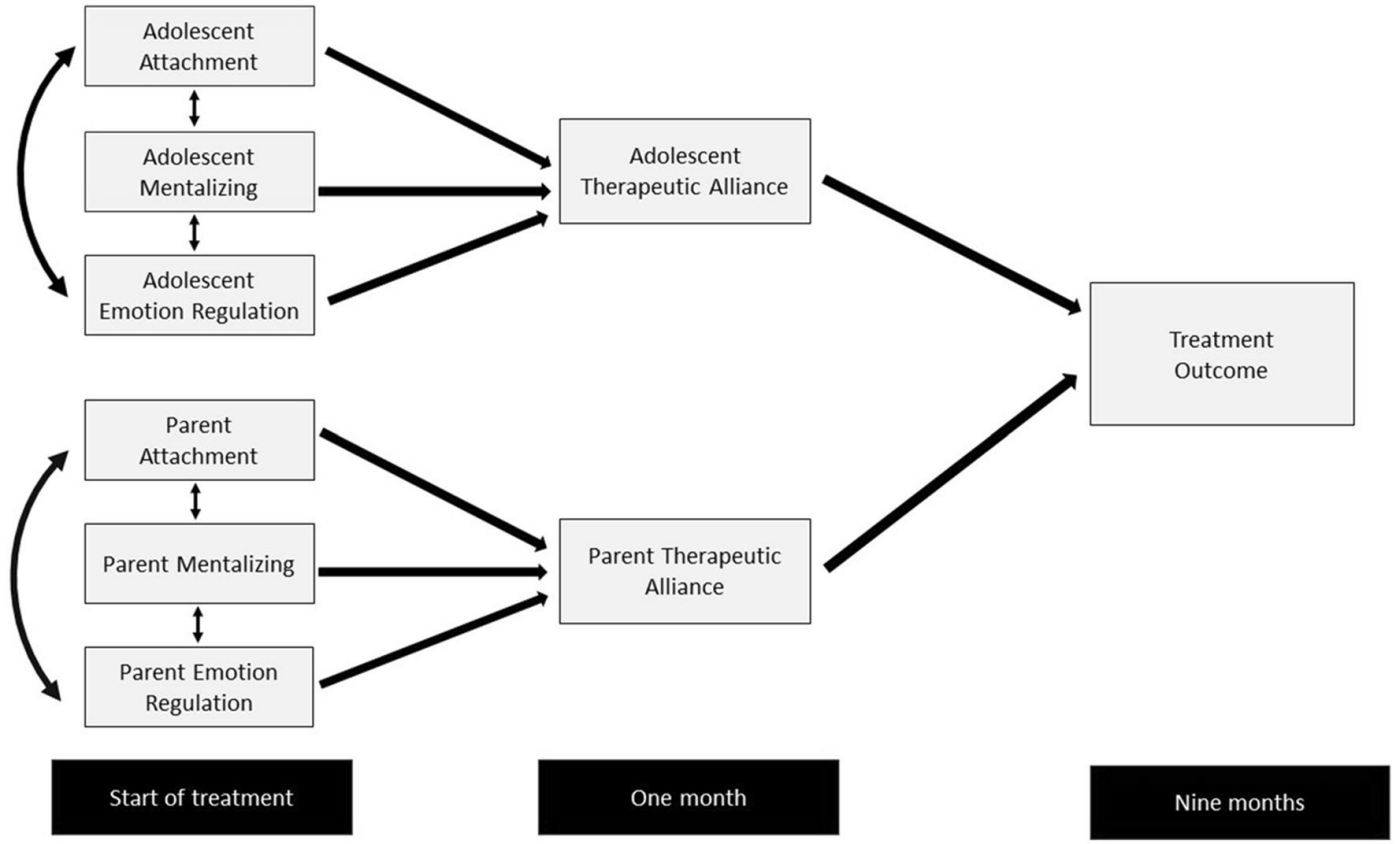
Conceptual diagram showing hypothesised model. Double headed arrows refer to correlations between variables

Attachment security was postulated as a relational strength that could increase the likelihood of forming a positive therapeutic alliance. By contrast, insecure attachment may make family members vulnerable to experiencing pre-mentalizing modes of thought in the context of emotionally charged situations. Within this model, emotional regulation might also emerge as a predictor of outcome, given its theoretical and empirical associations with attachment and mentalizing [25]. We proposed that insecure attachment, mentalizing difficulties, and emotional dysregulation at the start of treatment would be associated with poorer therapeutic alliance ratings at one month. We hypothesised that one mechanism by which attachment and mentalization might influence outcome is via their association with the development of therapeutic alliance, a well-established predictor of outcome in eating disorders and psychotherapy [26, 27]. Our theoretical model is depicted visually in Fig. 1.

#### Hypotheses

The aim of the study is to address the following hypotheses:

1. Baseline insecure attachment, mentalizing difficulties, and emotion regulation difficulties, in both adolescents and parents, will predict categorical poor treatment outcome at nine months.
2. Positive ratings of therapeutic alliance at one month by adolescents and parents will predict good categorical treatment outcome at nine months.
3. Secure attachment, better mentalizing, and lower emotion regulation difficulties at baseline will predict positive therapeutic alliance at one month for parents and adolescents.

### Secondary analysis

We will also conduct a secondary analysis to test the following hypothesis: insecure attachment, mentalizing difficulties, and emotion regulation difficulties, assessed at baseline in both adolescents and parents will predict lower gains in percentage median body mass index (%mBMI) at 9 months.

## Methods

Data were collected from three clinical sites in the United Kingdom, all of which are specialist community-based eating disorder services based in two diverse, multi-ethnic cities. Adolescent patients and their families meeting criteria for the study were approached at assessment or start of treatment. All consecutive cases meeting eligibility within the data collection period were invited to participate. Patient inclusion criteria were as follows: aged between 10 and 17; living with their parent/s or carer/s for at least the previous 3 months; diagnosed at clinical assessment with anorexia nervosa (restricting or binge/purge sub-type) using DSM-V criteria [28]; or meeting DSM-V criteria for Other Specified Feeding or Eating Disorder (OSFED) providing their % mBMI at assessment was at 85% or below, or the adolescent had lost 15% of their body weight in the 3 months prior to assessment; adequate level of English, i.e., sufficient to understand study information sheets and consent forms; receiving out-patient family therapy for anorexia nervosa (FT-AN) as their treatment. Parent/carer criteria were that they would be involved in attending FT-AN sessions and had an adequate level of English. At least one parent/carer needed to consent to take part in the study. Up to two parents/carers could be recruited per adolescent.

### Data collection

Self-report measures of attachment, mentalization, and emotion regulation were completed at the start of treatment (T1) and 1 month into treatment (T2) by adolescent patients and at least one of their parents. At T2 self-report measures of alliance were also completed by parents and adolescents.

Young people and parents completed self-report measures online using the Bristol Online Survey, usually from their own homes (approximately 5% of T1 questionnaires were completed on paper). Weight and height data were collected by clinicians as part of the routine clinical assessment. The conversions to percent median body mass index (%mBMI) were done using a computer programme based on the Child Growth Foundation [29] development charts.

### Outcomes

Primary outcome: Morgan–Russell scales as modified by Russell et al. [30] in which categorical outcome was recorded at 9 months at one of three levels, as follows:

*Good outcome*—participants whose weight is above 85%mBMI, who are menstruating and have no bulimic symptoms; *Intermediate outcome*—participants meet the same weight criteria, but are either not menstruating or have occasional bulimic symptoms (averaging less than once a week over the past month); *Poor outcome*—participants whose weight is below 85%mBMI or have developed bulimic symptoms more than once a week. Those in inpatient care at 9 months were also conservatively classified as having a poor outcome*.*

Weight data are reported as a percentage of median BMI (%mBMI), adjusted for height, age, and sex.

*Adolescent predictor variables at Time 1*

- Attachment Style Questionnaire (ASQ) [31]: This 40-item attachment measure was developed for use with both adults and adolescents and is comprised of five subscales: Confidence, Preoccupation with Relationships, Need for Approval, Relationships as Secondary, and Discomfort with Closeness. Internal reliability and test–retest reliability are both good.
- Difficulties in Emotion Regulation Strategies Scale (DERS) [32]: This 36-item measure assesses emotion regulation difficulties and is comprised of six subscales: Nonacceptance of Emotional Responses, Difficulties Engaging in Goal-directed Behaviours, Impulse Control Difficulties, Lack of Emotional Awareness, Limited Access to Effective Emotion Regulation Strategies, and Lack of Emotional Clarity. The measure has demonstrated high internal consistency and good construct and predictive validity in clinical and non-clinical populations.
- Hypermentalizing Questionnaire—Adolescent version (parent scale) (Sharp, Barr and Vanwoerden (2018) *Unpublished Manuscript*): This scale comprises 26 items which assess adolescent hypermentalizing in their interaction with parents (e.g., “I worry a lot about what my parents are thinking and feeling”). The measure yields a total Hypermentalizing score, in which higher scores represent greater levels of hypermentalizing. Validity for the scale comes from an unpublished study in which the measure demonstrated convergent validity with the hypermentalizing scale of the Movie for Assessing Social Cognition [33], an experimental measure of mentalizing. The measure has been validated in an adolescent psychiatric sample comprised predominantly of youths with a diagnosis of Borderline Personality Disorder.
- Reflective Function Questionnaire—Youth (RFQY) [34]: This 46-item measure yields a Total score, with higher scores representing greater reflective function (sample item: “I always know what I feel”). The RFQY has adequate internal reliability. Convergent, criterion, discriminant, and construct validity have been demonstrated in an adolescent inpatient psychiatric sample, of which a small minority of participants had an eating disorder.

*Parent predictor variables at Time 1*

- Reflective Function Questionnaire (RFQ8) [35]: This 8-item measure yields two subscales: Certainty about Mental States (henceforth referred to as Certainty) and Uncertainty about Mental States (henceforth: Uncertainty). Scoring is applied, such that extreme scores at either end of the Likert scale are indicative of inadequate mentalizing. For instance, strong agreement with the item “I always know what I feel” yields a higher score on the Certainty scale, whereas strong agreement with the item “I don’t always know why I do what I do” yield a higher score on the Uncertainty scale. The measure has demonstrated satisfactory levels of internal validity, convergent validity, and test–retest reliability.
- ASQ
- DERS
- Hypermentalizing Questionnaire—Parent version (adolescent scale): Parents completed the adolescent scale of the HMZ, which assesses parental hypermentalizing in their interaction with their adolescent (e.g., “My adolescent often says I over interpret his/her behavior or words”).

*Adolescent and parent Time 2 predictor variables*

- System for Observing Family Therapy Alliance—Self-report (SOFTA) [36]: This 16-item measure assesses perceptions of family therapy alliances on four subscales: Engagement in the Therapeutic Process, Emotional Connection to the Therapist; Safety within the Therapeutic System and Shared Sense of Purpose within the Family. Internal reliability for the total scale is good, and adequate-to-good for the four subscales. Convergent validity with an observer-rated version of the SOFTA has been demonstrated.

*Baseline covariates*

- Eating Disorder Examination Questionnaire (EDE-Q) [37]: This 28-item measure of eating disorder pathology has good internal consistency, test–retest reliability, and discriminant validity. The EDE-Q Global Score completed at initial clinical assessment was extracted from patient records.
- Baseline percentage median Body Mass Index (%mBMI) adjusted for age and gender.
- Duration of illness in months. This refers to the number of months that the young person had been unwell for prior to their assessment in clinic.
  EDE-Q was completed by adolescents at assessment (prior to entry to the study). Data on baseline percentage BMI and duration of illness were extracted from clinical notes from the time of clinical assessment.

*Baseline measures of co-morbidity*

- Revised Child Anxiety and Depression Scale (RCADS)—Youth self-report version [38]: This 47-item questionnaire has good-to-excellent internal consistency, good test–retest reliability, and strong convergent and divergent validity. The Total Internalising Scale completed at initial clinical assessment was extracted from patient records.
- Self-harm at assessment—this was based on adolescent report at clinical assessment and was extracted from patient records, coded as 1 (for present) or 0 (not present).

### Study registration

The study was pre-registered with Europe PubMed Central, with a brief overview of the study available at http://europepmc.org/grantfinder/grantdetails?query=pi%3A%22Jewell%20T%22%20gid%3A%22CDRF-2014-05-024%22%20ga%3A%22National%20Institute%20for%20Health%20Research%20%28NIHR%29%22&cat=.

### Ethics

The study was approved by the Camden and King’s Cross ethics committee of the Health Research Authority.

### Sample size

The study aimed to recruit 200 participants. The power calculation assumptions were that a sample size of 200 with follow-up of 80% at 9 months and 30% experiencing poor treatment outcome would have 80% power to detect an odds ratio (OR) of  > 1.58 in relation to a one standard deviation difference in the predictor variable in the logistic regression analysis with an alpha level of 0.05.

### Internal reliability

We report McDonald’s Omega for all self-report measures in Table 2. McDonald’s Omega was chosen since it is considered a less biased metric of internal consistency than Cronbach’s alpha. For the EDE-Q and RCADS data, we did not have item-level data available for our participants, as these scores were extracted from patient notes. Therefore, McDonald’s Omega for these two measures were calculated from routine outcome databases from Site 1.

### Public and patient involvement (PPI)

Parents and adolescents with lived experience of AN were involved in the design and interpretation of this study. The choice of measures and methods for approaching and recruiting participants were informed by PPI feedback. To guide interpretation, a public meeting was held in August 2019 to share findings with those with lived experience, which was followed up by dissemination over email of lay summaries in late 2019. The findings were discussed over email and telephone with several individuals with lived experience. Suggestions from these activities are included in the discussion section.

### Analysis plan

For the purposes of the main analysis, the primary outcome was a binary variable combining Good and Intermediate outcome to compare with Poor outcome on the Morgan Russell scales.

The analyses were conducted in two stages:

- 1. Binary logistic regression was used to test the association between predictors and therapy outcome at 9 months after treatment began.
- 2. Linear regression was used to test the associations between T1 and T2 predictors.

Robust standard errors were used in all analyses due to nonindependence of participants due to clustering by site. Alpha level was set at 0.05. Predictor variables were tested at the subscale level, with total scores used only when subscale scores were unavailable.

All analyses included the following covariates recorded at the time of clinical assessment: age (in years); duration of illness (in months) at the time of assessment; severity of eating pathology using the EDE-Q Global score; baseline percentage median Body Mass Index adjusted for age and gender; and treatment site.

### Missing data

Missing data for individual items on the self-report measures of predictor variables were low, since most questionnaires were completed online, with participants unable to submit their responses if items were missing. Approximately 5% of participants completed paper questionnaires at Time 1. Missing data were more frequent for Time 2 data. There were also higher rates of missing data for some baseline covariate measures, such as the RCADS and EDE-Q measures, which were drawn from data collected routinely in the clinics (see Table 1 for details). To minimise bias arising from complete case analysis, we used multiple imputation to impute missing values.

**Table 1:** Demographic characteristics of the sample

	*N*	Minimum	Maximum	Mean	SD
Age	173	11	17	14.69	1.54
% Median body mass index	173	62.27	113.79	83.89	8.97
Duration of illness (months)	170	1	65	10.48	10.59

	*N*	%
Sex		
Female	153	88.4
Male	20	11.6
Lives with		
Natural/adoptive parents	129	74.6
Mother alone	24	13.9
Other	20	11.6
Ethnicity		
White British	138	79.8
Other White	13	7.5
Dual Heritage	10	5.8
British Asian/Other South Asian	9	5.2
Other	3	1.7
Diagnosis		
Anorexia nervosa (restricting sub-type)	140	80.9
Anorexia nervosa (binge/purge sub-type)	9	5.2
Other Specified Feeding or Eating Disorder (OSFED)	24	13.9
Self-harm reported at assessment	35	20.2

### Multiple imputation

We analysed patterns of missingness for baseline covariates, Time 2 alliance scores, and percentage median body mass index at 9 months by creating new binary variables to indicate completeness/missingness for each variable of interest. Binary logistic regression was used to identify significant predictors of missingness. We used multiple imputation with chained equations [39] and entered the following significant predictors of missingness: the EDE-Q Global score; the RCADS Internalising total score; adolescent Attachment Style Questionnaire Confidence and Preoccupation scales; the adolescent Difficulties in Emotion Regulation Scale Total score; the Reflective Function Scale—Youth total score, and the parental Attachment Style Questionnaire Preoccupation Scale. Auxiliary variables were age, baseline percentage median body mass index, site, sex, outcome, and use of higher level of care.

We imputed missing baseline covariate, Time 2 alliance scores, and 9-month %mBMI median body mass index, using the MI impute chained command in Stata and 50 imputed datasets. All reported analyses of the primary and secondary outcome have been run using multiple imputation. All analyses were conducted in Stata v.15 (StataCorp, College Station, TX).

## Results

### Demographics

**Fig. 2:**
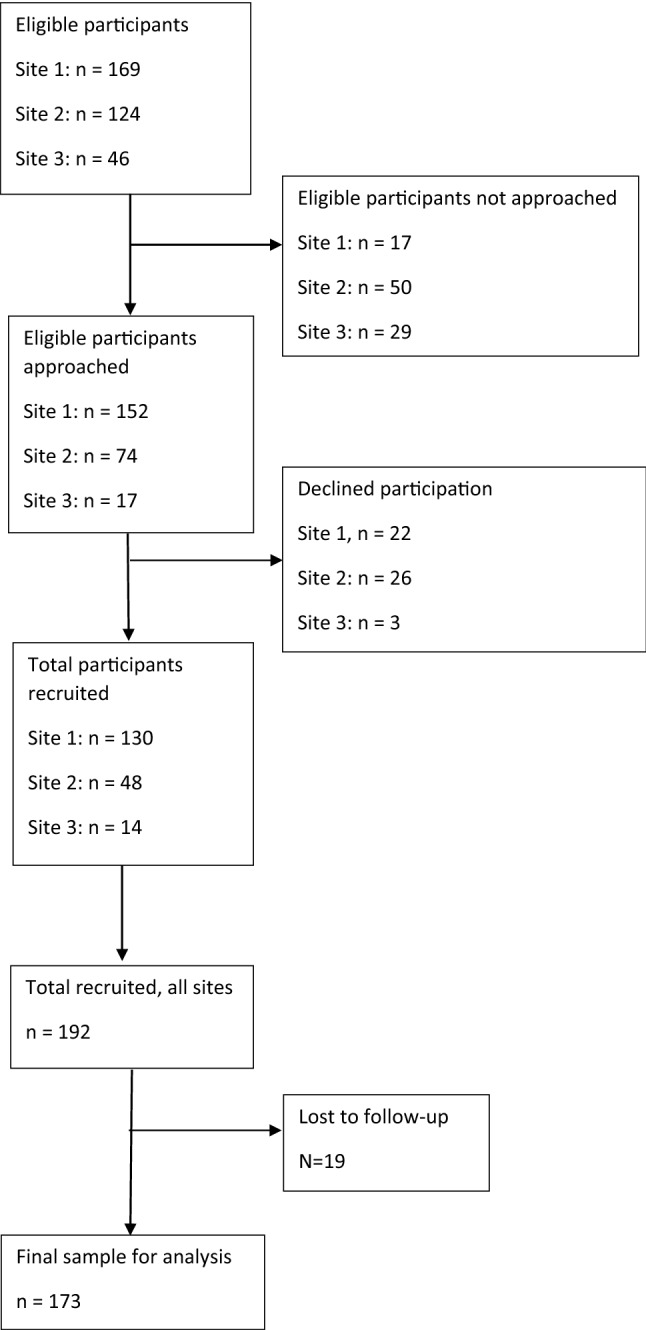
Recruitment flowchart

We recruited 192 adolescents and their parent/s to the study. Of this total, we excluded 19 participants (9.9% of the sample) for the following reasons: 12 participants did not complete any measures for the study; two participants withdrew their consent; four were recruited to the study in error without meeting inclusion criteria; one participant had baseline data but no outcome data. The recruitment flowchart for the study is presented in Fig. 2. Recruitment took place from September 2015 to July 2018 in Site 1; February 2016 to July 2018 in Site 2; and January 2017 to July 2018 in Site 3.

The final sample for whom baseline and outcome data were available comprised 173 adolescents, of whom 153 (88.4%) were female, with a mean age of 14.7 years. The parent sample comprised *n* = 163, of whom 14 parents were fathers and 149 parents were mothers. No parent data were available for ten adolescent participants. For 17 participants, data were available from both parents, as both had been recruited to the study. Since the majority of the parent sample were mothers, and to prevent artificially inflating the sample size beyond the 173 adolescents in the study, we excluded data from fathers in the 17 cases where mother data were available. Data from fathers were used for 14 adolescents who did not have data from mothers. 118 participants came from Site 1, 43 from Site 2, and 12 from Site 3. Demographic data for the sample are presented in Table 1. Thirty-four adolescents (19.7%) attended some form of higher level of care, including 18 (10.4%) who attended day-patient care, 11 (6.4%) who were admitted to inpatient psychiatric care, and 11 (6.2%) who were admitted to a paediatric ward.

### Correlations between predictor variables

Table S1 reports Pearson’s correlations between predictor variables.

### Descriptive statistics

The range, means, and standard deviations for self-report measures are presented in Table 2.

**Table 2:** Range, means, standard deviations, and internal reliability of self-report measures

Adolescents	*N*	Min	Max	Mean	SD	Omega
RCADS Total Internalising (T1)	127	3	136	55.21	27.33	0.97
EDE-Q Global (T1)	159	.00	5.70	3.35	1.56	0.95
ASQ Confidence (T1)	170	9	48	28.88	7.25	0.86
ASQ Discomfort (T1)	170	10	58	39.83	8.86	0.88
ASQ Secondary (T1)	170	7	38	19.59	5.94	0.80
ASQ need for approval (T1)	170	11	42	30.44	5.81	0.77
ASQ Preoccupation (T1)	170	8	46	29.82	6.70	0.76
DERS Nonacceptance (T1)	170	6	30	18.60	7.56	0.94
DERS Goals (T1)	170	5	25	17.98	5.20	0.89
DERS Impulse (T1)	170	6	30	17.62	6.82	0.92
DERS Awareness (T1)	170	6	30	17.62	5.10	0.81
DERS Strategies (T1)	170	8	40	25.78	8.62	0.93
DERS Clarity (T1)	170	5	25	14.58	4.98	0.89
HMZ (T1)	169	2	94	53.56	18.41	0.92
RFQY (T1)	170	6.26	10.44	8.78	.79	0.76
SOFTA engagement (T2)	125	4	20	12.36	3.62	0.75
SOFTA connection (T2)	125	4	20	14.34	4.00	0.84
SOFTA safety (T2)	125	4	20	11.52	3.70	0.70
SOFTA purpose (T2)	125	6	20	15.04	3.32	0.72

Parents	N	Min	Max	Mean	SD	Omega
ASQ Confidence (T1)	163	12	47	36.06	5.58	0.82
ASQ Discomfort (T1)	163	11	53	31.93	8.41	0.88
ASQ Secondary (T1)	163	7	28	14.57	4.62	0.72
ASQ need (T1)	162	7	36	20.73	5.60	0.78
ASQ Preoccupation (T1)	163	11	47	24.67	6.67	0.81
DERS Nonacceptance (T1)	163	6	30	11.42	5.14	0.88
DERS Goals (T1)	163	5	25	12.56	4.50	0.86
DERS Impulse (T1)	163	6	28	10.15	4.38	0.89
DERS Awareness (T1)	163	6	26	14.49	4.37	0.79
DERS Strategies (T1)	163	8	39	13.83	5.43	0.88
DERS Clarity (T1)	163	5	20	8.79	2.92	0.80
RFQ8 Certainty (T1)	163	.00	3.00	1.22	.74	0.79
RFQ8 Uncertainty (T1)	163	.00	2.17	.41	.45	0.76
HMZ (T1)	162	8	81	39.78	17.55	0.93
SOFTA Engagement (T2)	132	8	20	15.86	2.65	0.67
SOFTA Connection (T2)	132	7	20	15.77	2.83	0.77
SOFTA Safety (T2)	132	8	20	15.97	2.76	0.66
SOFTA Purpose (T2)	132	8	20	16.83	2.85	0.75

*RCADS* Revised Child Anxiety and Depression Scale (RCADS); *EDE-Q* Eating Disorder Examination Questionnaire; *ASQ* Attachment Style Questionnaire; *ASQ Discomfort* ASQ Discomfort with Closeness; *ASQ Preoccupation* ASQ Preoccupation with Relationships; *ASQ Secondary* ASQ Relationships as Secondary; *DERS* Difficulties in Emotion Regulation Strategies Scale; *DERS Nonacceptance* DERS Nonacceptance of Emotional Responses; *DERS Goals* DERS Difficulties Engaging in Goal-Directed Behaviours; *DERS Impulse* DERS Impulse Control Difficulties; *DERS Awareness* DERS Lack of Emotional Awareness; *DERS Strategies* DERS Limited Access to Effective Emotion Regulation Strategies; *DERS Clarity* DERS Lack of Emotional Clarity; *RFQY* Reflective Function Questionnaire – Youth Total Score; *HMZ* Hypermentalizing Questionnaire; *SOFTA* System for Observing Family Therapy Alliance; *SOFTA Engagement* SOFTA Engagement in the Therapeutic Process; *SOFTA Connection* SOFTA Emotional Connection to the Therapist; *SOFTA Safety* SOFTA Safety within the Therapeutic System; *SOFTA Purpose* SOFTA Shared Sense of Purpose within the Family; *RFQ8* Reflective Function Questionnaire (8-item version); *RFQ8 Certainty* RFQ8 Certainty About Mental States; *RFQ8 Uncertainty* RFQ8 Uncertainty About Mental States. Omega refers to McDonald’s Omega

### Prediction of outcome from baseline covariates

A higher baseline %mBMI (OR = 1.11, CI: 1.04–1.18) and higher self-reported eating pathology (EDE-Q Global score) (OR = 1.36, CI: 1.01–1.83) were associated with higher odds of positive outcome. Older age (OR: 0.63, CI: 0.48–0.82) and longer duration of illness (OR: 0.97, CI: 0.94–1.00) were associated with higher odds of poor outcome.

### Associations between predictors and outcome

The associations between all predictor variables with treatment outcome, as well as associations between all T1 and T2 predictors, are available in Tables S2 and S3 for parents and adolescents, respectively. In the following sections, we highlight the significant findings for each pathway.

### Prediction of outcome from baseline variables

Of the parent measures, higher scores on the RFQ8 Certainty subscale were predictive of poor outcome (OR = 0.42, CI: 0.20–0.87), whilst higher scores on the DERS Impulsive subscale (OR = 1.23, CI: 1.07–1.43) and Hypermentalizing Questionnaire (OR = 1.03, CI: 1.00–1.06) predicted positive outcome. Of the adolescent measures, higher scores on the DERS Lack of Clarity scale, in which higher scores represent being unclear about one’s feelings, predicted positive outcome (OR = 1.10, CI: 1.00–1.21).

### Prediction of outcome by alliance

Of the SOFTA subscales, positive outcome was predicted by parental Emotional Connection score (OR = 1.32, CI: 1.09–1.58), parental Sense of Safety score (OR = 1.24, CI: 1.02–1.50), and the adolescent Shared Sense of Purpose score (OR = 1.18, CI: 1.01–1.38).

### Prediction of time 2 alliance by time 1 predictor variables

Of the SOFTA subscales that were predictive of outcome, parental SOFTA Sense of Safety scores were predicted by parental ASQ Confidence (β = 0.17, CI: 0.07–0.26), parental ASQ Preoccupation (β =  − 0.11, CI: − 0.19 to  − 0.03), parental ASQ Need for Approval (β =  − 0.10, CI: − 0.19 to  − 0.01), and parental DERS Goals subscale (β =  − 0.15, CI: − 0.26 to  − 0.03). No parental Time 1 predictor variables predicted parental SOFTA Emotional Connection scores.

For adolescents, the SOFTA Shared Sense of Purpose score was predicted by the adolescent DERS Awareness subscale (β =  − 0.15, CI: − 0.28 to  − 0.02) and RFQY score (β = 1.02, CI: 0.19–1.85).

### Secondary analysis: prediction of percentage median body mass index at 9 months

No parent or adolescent predictor variables predicted %mBMI at 9 months.

## Discussion

Our study investigated the hypothesis that attachment and mentalizing would predict alliance and outcome in FT–AN. Whilst the alliance was predictive of outcome in our data, and attachment and mentalizing were predictive of alliance, our theoretical model was only partially supported. Our results suggest that excessive certainty about mental states, a variable uncorrelated with alliance, was the strongest predictor of poor outcome, with each unit increase on the RFQ8 Certainty scale reducing the odds of a good outcome by more than half. For adolescents, higher scores on the DERS Lack of Clarity subscale also emerged as a predictor of outcome. Whilst this finding was in the opposite direction to our hypotheses, it is consistent with our findings for parents, since extreme clarity about one’s feelings is conceptually similar to certainty about mental states, and indeed, we found that parental RFQ8 Certainty and adolescent DERS Lack of Emotional Clarity scores are negatively correlated in our data (*r* =  − 0.28, *p* < 0.01). In terms of possible mediating mechanisms, we speculate that excessive certainty might correlate with rigidity and difficulties in considering alternative perspectives. This could hamper recovery through reliance on a limited repertoire of problem-solving approaches.

In our PPI consultation, it was suggested that a high sense of clarity about one’s feelings might be associated with a sense of certainty deriving from the eating disorder, and perhaps an identification with the ‘eating disorder voice’ which is associated with markers of severity such as longer duration of illness [40]. This perspective is congruent with the notion that ineffective mentalizing might influence outcome through lower levels of epistemic trust [41], resulting in reduced openness to learning in therapy. Relatedly, within the family context, excessive certainty on the part of the adolescent might also hamper their openness to accepting support from parents and might lead to a circular process whereby parental attempts to promote change are met with increased psychological rigidity on the part of the adolescent.

The association between outcome and the parental SOFTA Emotional Connection and Safety scales is in keeping with themes from qualitative studies which emphasise the importance for parents of trusting and feeling understood by their therapist [42, 43]. Wallis et al. [42] have described a therapeutic process in FT-AN that they term *relational containment*, whereby treatment factors including the structured nature of the treatment, specialist treatment expertise, and consistent support create a sense of stability for parents and an expectation of progress, which helps to give them the confidence to validate their child’s experiences and, at the same time, provide a supportive, predictable environment conducive to change. This then helps to establish a virtuous circle whereby increased parental confidence helps their adolescent to feel more secure, thereby promoting positive changes in eating behaviours as well as improved communication. This is consistent with our finding that the adolescent ratings on the SOFTA Shared Sense of Purpose were also predictive of a positive outcome. Our findings support the notion that the development of this sense of relational containment in the family may represent an important early treatment goal, which, from the perspective of adolescents, might contribute to establishing a shared sense of purpose for therapy. Notably, secure attachment and higher reflective function in adolescents were both predictive of positive alliance ratings, suggesting that these variables merit inclusion in future studies of alliance formation in FT–AN, such as studies incorporating measures of social and work functioning, session-by-session data collection, and observational measures of alliance.

Our study has several limitations, such as the use of self-report methods for both Time 1 and Time 2 predictors, thereby raising the issue of single-method variance. Furthermore, our measures of mentalization are relatively new, and have not yet been validated in adolescent eating disorder samples. Feedback from PPI activities highlighted that our choice of outcome was too reliant on weight as a criterion for success and did not include broader themes, such as positive indicators of well-being, which have been shown to be important to recovery from the perspective of those with lived experience [44].

## Conclusion

In summary, our data are the first to suggest that mentalizing difficulties may predict poor outcome in FT-AN, particularly when present in parents. Study strengths include the relatively large sample drawn from three different sites providing specialist out-patient treatment, thereby increasing the external validity of the study. Other strengths include the use of PPI to inform both method and interpretation. Future research should use multi-method approaches to investigate processes of change, including the roles of mentalizing and alliance, in studies using broader definitions of recovery that are salient to parents and adolescents with lived experience of eating disorders.

## Supplementary Information

Below is the link to the electronic supplementary material.

Supplementary file1 (XLSX 48 KB)

Supplementary file2 (DOCX 26 KB)

Supplementary file3 (DOCX 28 KB)

## Data Availability

Data are available from the corresponding author on request.

## Code availability

Code is available from the corresponding author on request.
