# Supplementary material for: Attachment and mentalization as predictors of outcome in family therapy for adolescent anorexia nervosa
Source: Eur Child Adolesc Psychiatry. 2021 Dec 30;32(7):1241–51. doi: 10.1007/s00787-021-01930-3 (PMC10276078; doi:10.1007/s00787-021-01930-3)
Supplement: Supplementary file 2 — Supplementary file2 (DOCX 26 KB) [file 787_2021_1930_MOESM2_ESM.docx]

|  | **Parent Predictors T2** | | | | | | | | **Outcome at end of treatment** | | |
| --- | --- | --- | --- | --- | --- | --- | --- | --- | --- | --- | --- |
| **Parent Predictors** | **SOFTA Engagement** | | **SOFTA Connection** | | **SOFTA Safety** | | **SOFTA Purpose** | | **Morgan Russell**  **Outcome** | | |
| **T1** | **Beta** | **95% CI** | **Beta** | **95% CI** | **Beta** | **95% CI** | **Beta** | **95% CI** | **OR** | **95% CI** | |
| ASQ Confidence | .06 | −.03 – .16 | .08 | −.02 – .18 | .17 | .07 – .26 | .08 | −.01 – .18 | 1.02 | .95 – 1.11 |  |
| ASQ Discomfort | .00 | −.06 – .07 | .01 | −.06 – .07 | −.06 | −.12 – .01 | −.03 | −.09 – .02 | 1.01 | .95 – 1.08 |  |
| ASQ Secondary | −.06 | −.18 – .05 | –.06 | −.18 – .07 | −.10 | −.21 – .01 | −.11 | −.24 – .02 | 1.02 | .91 – 1.14 |  |
| ASQ Need for Approval | −.01 | −.10 – .08 | .03 | −.07 – .13 | −.10 | −.19 – −.01 | −.09 | −.18 – .00 | 1.04 | .94 – 1.15 |  |
| ASQ Preoccupation | −.02 | −.10 – .06 | –.02 | −.10 – .07 | −.11 | −.19 – −.03 | −.08 | −.15 – −.00 | 1.03 | .96 – 1.10 |  |
| DERS Nonacceptance | −.00 | −.10 – .09 | .02 | −.08 – .13 | −.08 | −.18 – .02 | −.09 | −.19 – .02 | 1.09 | 1.00 – 1.19 |  |
| DERS Goals | −.02 | −.14 – .10 | –.03 | −.15 – .09 | −.15 | −.26 – −.03 | −.13 | −.25 – −.02 | 1.07 | .96 – 1.19 |  |
| DERS Impulse | −.01 | −.13 – .11 | .00 | −.13 – .13 | −.05 | −.17 – .08 | −.12 | −.26 – .01 | 1.23 | 1.07 – 1.43 |  |
| DERS Awareness | −.03 | −.13 – .08 | .04 | −.07 – .15 | −.07 | −.17 – .04 | −.04 | −.15 – .08 | .93 | .83 – 1.05 |  |
| DERS Strategies | −.00 | −.10 – .09 | .00 | −.10 – .11 | −.10 | −.21 – .01 | −.08 | −.19 – .03 | 1.04 | .94 – 1.15 |  |
| DERS Clarity | .01 | −.17 – .17 | .07 | −.11 – .25 | −.13 | −.31 – .04 | −.22 | −.38 – −.04 | 1.15 | .97 – 1.38 |  |
| HMZ | −.01 | −.04 – .02 | .00 | −.03 – .03 | −.03 | −.05 – .00 | −.04 | −.07 – −.01 | 1.03 | 1.00 – 1.06 |  |
| RFQ8 Certainty | .16 | −.50 – .81 | –.13 | −.84 – .58 | .28 | −.40 – .96 | .93 | .24 – 1.62 | .42 | .20 – .87 |  |
| RFQ8 Uncertainty | −.02 | −1.14 – 1.10 | .62 | −.54 – 1.78 | −.81 | −1.95 – .32 | −1.13 | −2.32 – .05 | 2.72 | .78 – 9.51 |  |
| **T2** |  |  |  |  |  |  |  |  |  |  |  |
| SOFTA Engagement | – | – | – | – | – | – | – | – | 1.17 | .97 – 1.40 |  |
| SOFTA Connection | – | – | – | – | – | – | – | – | 1.32 | 1.09 – 1.58 |  |
| SOFTA Safety | – | – | – | – | – | – | – | – | 1.24 | 1.02 – 1.50 |  |
| SOFTA Purpose | – | – | – | – | – | – | – | – | 1.07 | .90 – 1.26 |  |

**Table S2: Associations between parent T1 and T2 predictor variables and outcome.** All analyses included the following covariates: age, percentage median BMI at baseline, self–reported eating disorder pathology, duration of illness and site. Abbreviations: CI, Confidence Interval; OR, Odds Ratio; ASQ, Attachment Style Questionnaire; ASQ Discomfort, ASQ Discomfort with Closeness; ASQ Preoccupation, ASQ Preoccupation with Relationships; ASQ Secondary, ASQ Relationships as Secondary; DERS, Difficulties in Emotion Regulation Strategies Scale; DERS Non–acceptance, DERS Non-Acceptance of Emotional Responses; DERS Goals, DERS Difficulties Engaging in Goal-Directed Behaviors; DERS Impulse, DERS Impulse Control Difficulties; DERS Awareness, DERS Lack of Emotional Awareness; DERS Strategies, DERS Limited Access to Effective Emotion Regulation Strategies; DERS Clarity, DERS Lack of Emotional Clarity; HMZ, Hypermentalizing Questionnaire; RFQ8, Reflective Function Questionnaire (8 item version); RFQ8 Certainty, RFQ8 Certainty About Mental States; RFQ8 Uncertainty, RFQ8 Uncertainty About Mental States; SOFTA, System for Observing Family Therapy Alliance; SOFTA Engagement, SOFTA Engagement in the Therapeutic Process; SOFTA Connection, SOFTA Emotional Connection to the Therapist; SOFTA Safety, SOFTA Safety within the Therapeutic System; SOFTA Purpose, SOFTA Shared Sense of Purpose within the Family.
