# Supplementary material for: Attachment and mentalization as predictors of outcome in family therapy for adolescent anorexia nervosa
Source: Eur Child Adolesc Psychiatry. 2021 Dec 30;32(7):1241–51. doi: 10.1007/s00787-021-01930-3 (PMC10276078; doi:10.1007/s00787-021-01930-3)
Supplement: Supplementary file 3 — Supplementary file3 (DOCX 28 KB) [file 787_2021_1930_MOESM3_ESM.docx]

|  | **Adolescent Predictors T2** | | | | | | | | **Outcome at end of treatment** | | |
| --- | --- | --- | --- | --- | --- | --- | --- | --- | --- | --- | --- |
| **Adolescent Predictors** | **SOFTA Engagement** | | **SOFTA Connection** | | **SOFTA Safety** | | **SOFTA Purpose** | | **Morgan Russell**  **Outcome** | | |
| **T1** | **Beta** | **95% CI** | **Beta** | **95% CI** | **Beta** | **95% CI** | **Beta** | **95% CI** | **OR** | **95% CI** | |
| ASQ Confidence | .14 | .04 – .24 | .06 | −.06 – .17 | .13 | .03 – .24 | .08 | −.00 – .16 | 1.00 | .93 – 1.07 |  |
| ASQ Discomfort | −.08 | −.16 – .00 | −.03 | −.13 – .07 | −.07 | −.16 – .02 | −.03 | −.10 – .03 | 1.00 | .95 – 1.06 |  |
| ASQ Secondary | −.10 | −.22 – .02 | −.08 | −.22 – .06 | −.09 | −.22 – .05 | −.04 | −.15 – .06 | .99 | .92 – 1.08 |  |
| ASQ Need for Approval | .01 | −.11 – .14 | .14 | −.01 – .28 | −.05 | −.19 – .08 | .01 | −.11 – .12 | 1.00 | .92 – 1.09 |  |
| ASQ Preoccupation | −.03 | −.15 – .09 | .06 | −.08 – .19 | −.01 | −.13 – .12 | −.07 | −.18 – .03 | 1.01 | .94 – 1.10 |  |
| DERS Nonacceptance | .03 | −.07 – .12 | .07 | −.04 – .18 | −.01 | −.12 – .09 | .02 | −.07 – .11 | 1.02 | .95 – 1.09 |  |
| DERS Goals | −.01 | −.15 – .14 | .08 | −.08 – .25 | −.03 | −.19 – .13 | .01 | −.13 – .14 | .96 | .88 – 1.06 |  |
| DERS Impulse | −.07 | −.18 – .04 | .02 | −.09 – .14 | −.10 | −.21 – .02 | −.04 | −.14 – .05 | 1.01 | .94 – 1.09 |  |
| DERS Awareness | −.22 | −.35 – −.08 | −.19 | −.35 – −.03 | −.16 | −.31 – −.02 | −.15 | −.28 – −.02 | 1.01 | .92 – 1.11 |  |
| DERS Strategies | −.06 | −.14 – .03 | .01 | −.10 – .11 | −.10 | −.20 – .00 | −.06 | −.14 – .02 | .97 | .91 – 1.03 |  |
| DERS Clarity | −.05 | −.19 – .10 | .03 | −.14 – .20 | −.07 | −.23 – .09 | −.06 | −.19 – .06 | 1.10 | 1.00 – 1.21 |  |
| HMZ | −.03 | −.07 – .02 | .01 | −.04 – .06 | −.04 | −.09 – .00 | −.02 | −.06 – .02 | 1.01 | .99 – 1.04 |  |
| RFQY | 1.10 | .14 – 2.07 | 1.33 | .19 – 2.47 | 1.05 | .02 – 2.07 | 1.02 | .19 – 1.85 | 1.15 | .68 – 1.94 |  |
| **T2** |  |  |  |  |  |  |  |  |  |  |  |
| SOFTA Engagement | – | – | – | – | – | – | – | – | 1.11 | .96 – 1.27 |  |
| SOFTA Connection | – | – | – | – | – | – | – | – | 1.09 | .96 – 1.24 |  |
| SOFTA Safety | – | – | – | – | – | – | – | – | 1.08 | .93 – 1.26 |  |
| SOFTA Purpose | – | – | – | – | – | – | – | – | 1.18 | 1.01 – 1.38 |  |

**Table S3: Associations between adolescent T1 and T2 predictor variables and outcome.**

All analyses included the following covariates: age, percentage median BMI at baseline, self–reported eating disorder pathology, duration of illness and site. Abbreviations: CI, Confidence Interval; OR, Odds Ratio; ASQ, Attachment Style Questionnaire; ASQ Discomfort, ASQ Discomfort with Closeness; ASQ Preoccupation, ASQ Preoccupation with Relationships; ASQ Secondary, ASQ Relationships as Secondary; DERS, Difficulties in Emotion Regulation Strategies Scale; DERS Nonacceptance, DERS Nonacceptance of Emotional Responses; DERS Goals, DERS Difficulties Engaging in Goal-Directed Behaviors; DERS Impulse, DERS Impulse Control Difficulties; DERS Awareness, DERS Lack of Emotional Awareness; DERS Strategies, DERS Limited Access to Effective Emotion Regulation Strategies; DERS Clarity, DERS Lack of Emotional Clarity; HMZ, Hypermentalizing Questionnaire; RFQY, Reflective Function Questionnaire – Youth; SOFTA, System for Observing Family Therapy Alliance; SOFTA Engagement, SOFTA Engagement in the Therapeutic Process; SOFTA Connection, SOFTA Emotional Connection to the Therapist; SOFTA Safety, SOFTA Safety within the Therapeutic System; SOFTA Purpose, SOFTA Shared Sense of Purpose within the Family.
